# Supplementary figures and images for: APC2 is critical for ovarian WNT signalling control, fertility and tumour suppression
Source: BMC Cancer. 2019 Jul 10;19:677. doi: 10.1186/s12885-019-5867-y (PMC6617595; doi:10.1186/s12885-019-5867-y)

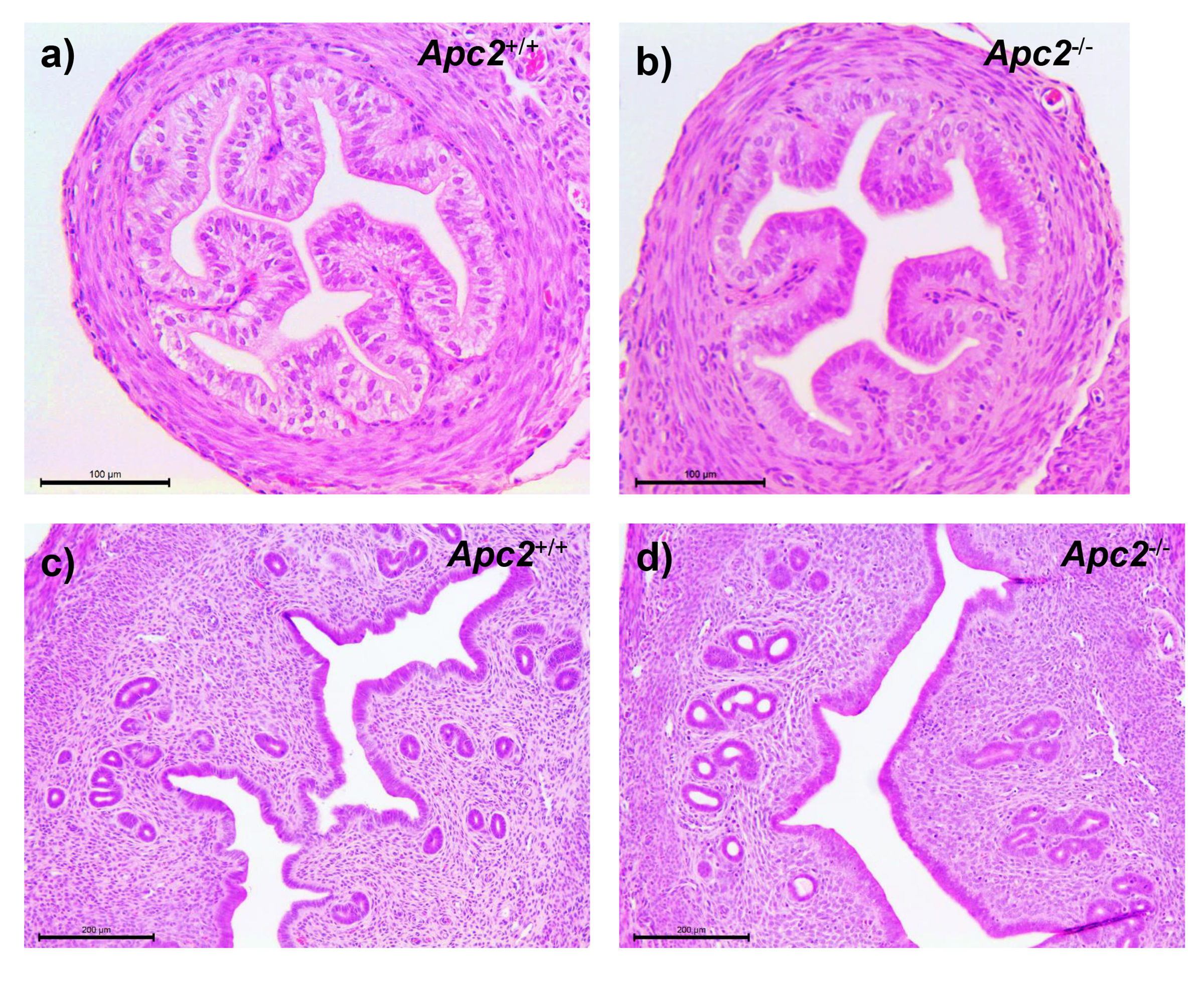

Supplement: Supplementary file 2 — Figure S1. APC2 is dispensable for oviduct and uterine gross morphology (.tiff). (TIF 8248 kb) [file 12885_2019_5867_MOESM2_ESM.tif]

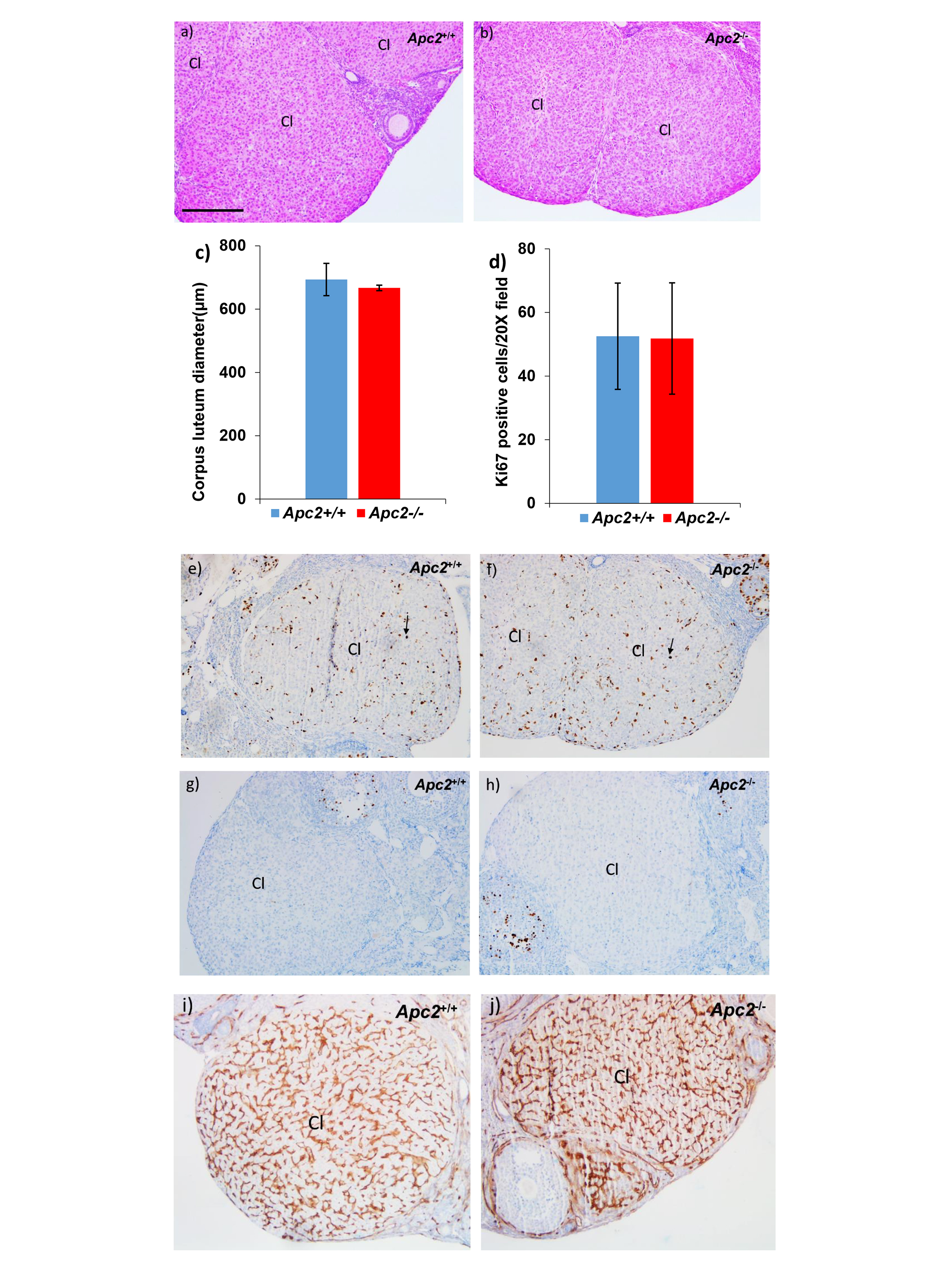

Supplement: Supplementary file 3 — Figure S2. APC2 is dispensable for corpora lutea (.tiff). (TIF 6547 kb) [file 12885_2019_5867_MOESM3_ESM.tif]

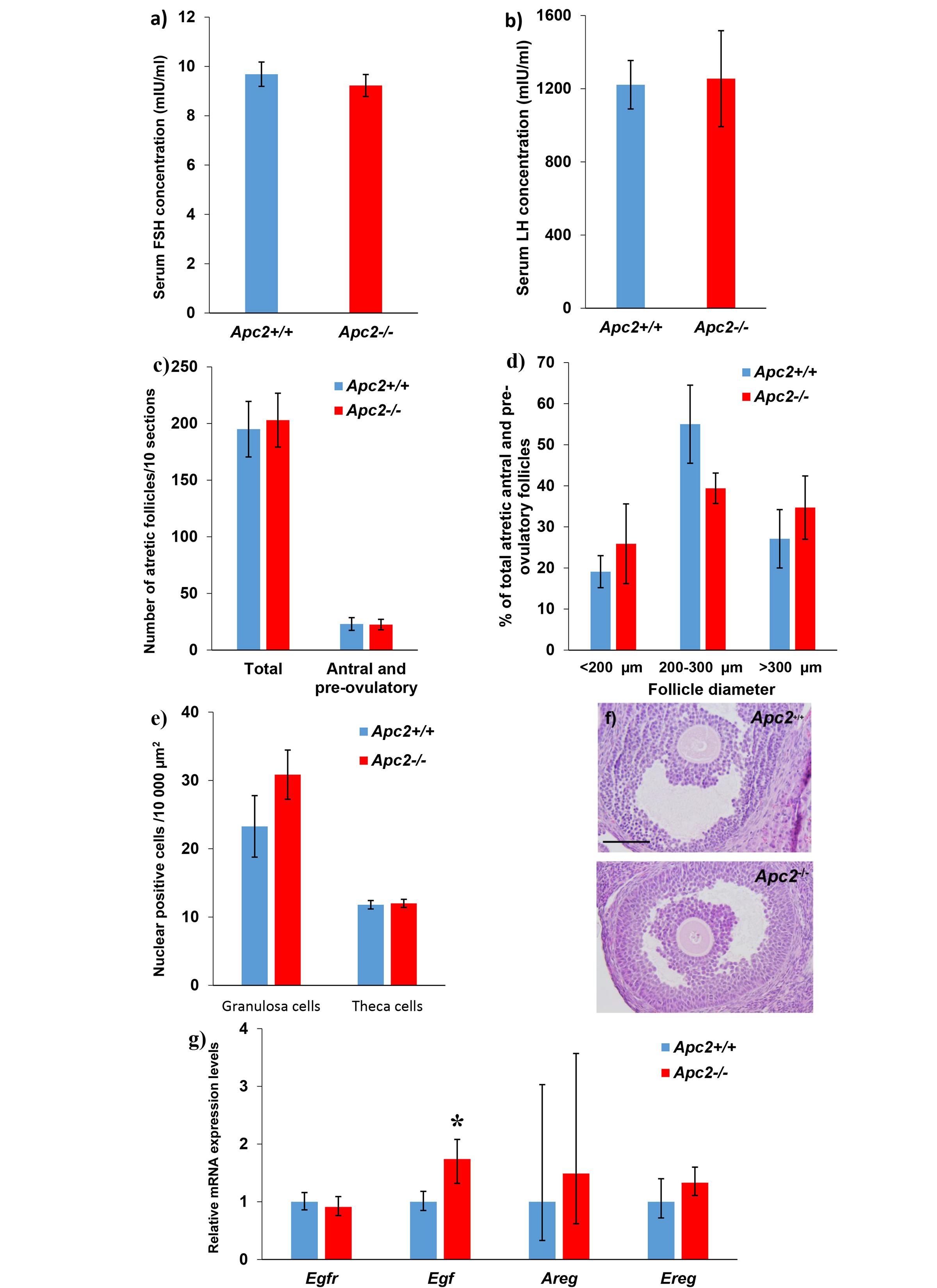

Supplement: Supplementary file 4 — Figure S3. Constitutive loss of APC2 has no effect on fertility hormones produced by pituitary gland or on the ovulation process (.tiff). (TIF 1633 kb) [file 12885_2019_5867_MOESM4_ESM.tif]

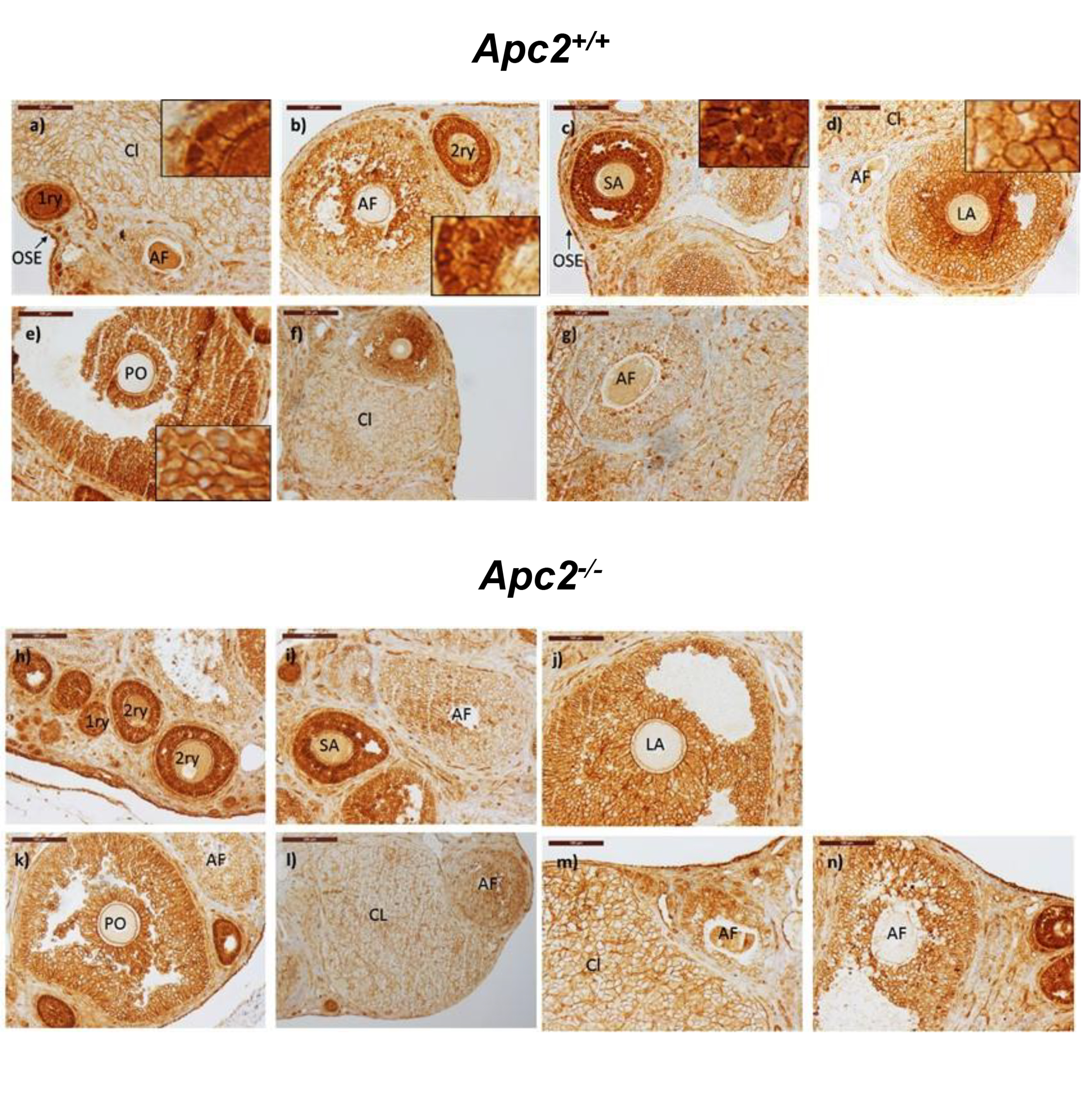

Supplement: Supplementary file 5 — Figure S4. Immunohistochemical localization of β-catenin protein in ovaries (.tiff). (TIF 6110 kb) [file 12885_2019_5867_MOESM5_ESM.tif]

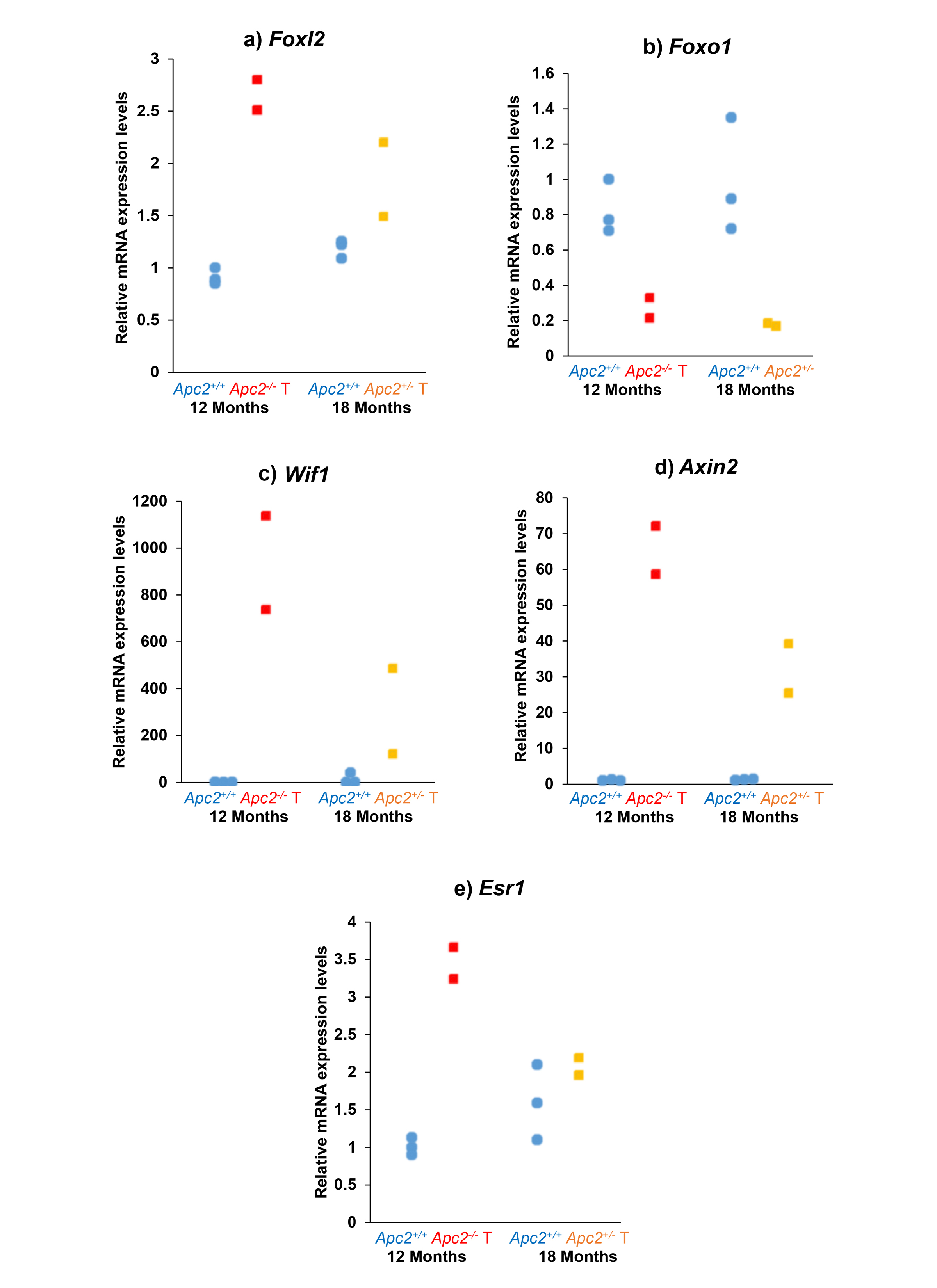

Supplement: Supplementary file 6 — Figure S5. Gene expression analysis of GCTs formed in a subset of APC2-deficient ovaries (.tiff). (TIF 705 kb) [file 12885_2019_5867_MOESM6_ESM.tif]
